# Mental Derangement

**Published:** 1827-07

**Authors:** 


### MEDICO-CHIRURGICAL REVIEW.

(THIRD NUMBER OF 1827.)

"Nec tibi quid liceat sed quid fecisse decebit
"Occurrat, mentemque domat respectus honesti."

VOL. VII.] JULY 1, 1827. [No. 13.

[NEW SERIES.]

### I.

### MENTAL DERANGEMENT.

1. *Des Causes Morales et Physiques des Maladies Mentales, et des quelques autres Affections Nerveuses, &c.* Par F. VOISIN, M.D.

*The Moral and Physical Causes of Mental Diseases and other Nervous Affections, as Hysteria, Nymphomania, Satyriasis, &c.*

2. *Observations on the Causes, Symptoms, and Treatment of Derangement of the Mind, founded on an extensive Moral and Medical Practice in the Treatment of Lunatics.* By PAUL SLADE KNIGHT, M.D. many years Surgeon of the Lunatic Asylum for the County of Lancaster. Octavo, pp. 166, with Plates. 1826.

MAN being of a compound nature, a rational soul inhabiting a material fabric, we apprehend that the moral philosopher has studied the mental faculties without sufficient reference to the body—while the physician has looked too exclusively to physical phenomena, and especially to physical causes as producing bodily disease. Yet the manifestations of mind are not more influenced by the state of the corporeal organs, than are the bodily functions by the state of the mind. It is on account of this wonderful and inexplicable connexion between mind and matter, that we rarely see, in high states of civilization, the simple effects of either moral or physical causes of disease. The moment an effect is produced by either class of agents, it becomes a *cause* in its turn, and thus action and re-action are continually going on between the mental and corporeal functions. The loss of a relation or the failure of a speculation will disorder the stomach through the medium of the mind—or improper food will disorder the mind through the medium of the stomach. Here the brain and stomach act and re-act upon each other, producing a complication of phenomena not so easily unravelled as some people imagine. And if we consider the number of organs in the body continually sympathising with each other, and the host of moral and physical causes which are daily calling these sympathies into play, we need hardly wonder at the interminable list of indescribable maladies, intellectual and corporeal, which is presented to the medical eye at every step in civilized life.

That aberrations of the intellect have increased in modern times—or at least in proportion as civilization has advanced, there can be as little doubt, as that the brain is more exercised in refined than in savage life. But the causes, the seat, and the nature of mental alienation are far from being completely investigated, or thoroughly understood. The author of the first work now before us comes forward to prove that insanity is an *idiopathic* affection of the brain, resulting from causes that act *directly* on that organ—or, in other words, that mental alienation is produced almost entirely by too violent or long-continued exercise of the cerebral functions. It is his object to shew that various physical causes, such as suppression of the menses, accouchments, critical periods of life, &c. which have been regarded by authors as powerfully productive of insanity, do *not* occasion that malady—that, on the contrary, we have, in all such cases, mistaken the effect for the cause—that the disordered function of the uterus, for example, is consecutive to a moral affection. He does not, indeed, deny that the brain sympathises with various other organs of the body; but he maintains that, in the great majority of cases where the functions of the brain are thus disordered sympathetically, we have confounded *acute delirium* with mental alienation, which last is a primitive or idiopathic affection of the brain.

In this place our author guards against misinterpretation of his doctrine.

"When I say that the brain is the material organ (he expresses it, the *material condition*) of intellectual faculties and moral qualities, I have nothing to fear from the system of interpretations, (le système des interprétations.) The muscles and the bones are the organs (conditions) of motion, but they are not the faculty which causes the motion. The eye is the organ of light, but is not the faculty of vision. I believe in the immateriality and immortality of the soul; but, so long as this last is united to the body, it requires corporeal instruments for its manifestation; and these manifestations are modified, diminished, augmented, or deranged, by the disposition of these instruments."

This is the conclusion to which all rational physiologists and metaphysicians must come in the end. It is precisely the language employed by Charron (de sagesse) two hundred years ago, who concludes a passage in these words:—" aussi l'esprit selon la diversité des dispositions organiques, des instrumens corporels, raisonne mieux, ou moins. Or l'instrument de l'âme raisonnable, c'est le cerveau."—*Liv.* 1, *p.* 88.

The fashionable modification of this doctrine which lately made so much noise on both sides of the channel—namely, that the sum total of the organic functions *was* the soul, (though couched under the term *life*,) is now on its wane, even in France; but, whether from the fear of the priests, the eagle eyes of the press censors, or the prevalence of a more enlightened philosophy, we are unable to determine.

I. The subject of education first engages our author's attention, and certainly the investigation of its influence on the happiness of mankind is well deserving of serious consideration. He does not accord with Locke, (nor indeed can any physiologist agree with this celebrated metaphysician,) that the brain is a blank tablet at first, on which all kinds of characters may be engraved. Man is not a passive being, equally susceptible of all impressions. His *dispositions* are innate, and it is not to accidental impressions received through the medium of the senses, or furnished by education, that he is indebted for those imperious propensities, profound sentiments, and remarkable talents, that have placed him at the summit of the scale of animated beings. " Each individual, in virtue of his organization, has his own peculiar character. Nature has lavished her favours on some people, and behaved most miserly to others." This inequality, our author contends, is in perfect harmony with the inequality of organization. Nevertheless, education has great influence in giving scope and direction to talents and moral qualities on the one hand, and in giving assistance to feeble innate powers on the other. Education has also great power in checking evil, and cherishing good innate qualities; though it cannot *create* either of these.—Esquirol, who seems to attribute too much to the influence of education, and too little to that of innate disposition, or, in other words, to organization, has acknowledged that the great majority of the insane, who came under his cognizance, had evinced signs of eccentricity long before the period of actual alienation. Some were proud, even from their infancy; others choleric—some were melancholic; others volatile, even to a ridiculous excess. It is probably true, as our author avers, that all those men who have astonished the world by the variety of their genius, the elevation of their souls, or the extent of their wickedness, have evinced signs of what was to break forth, from their earliest infancy, and long before education could have exerted a benign or malignant influence on their minds. Dr. Gall has observed that education, instruction, example, and other accidental circumstances, are most influential where the innate dispositions are neither too feeble nor too energetic. All men, says he, of medium innate powers, or organization, have *capacity* for those things which are proper and necessary for the situation in which man is usually placed. This medium is what Nature has aimed at generally. With this mediocrity of moral and intellectual powers, men may be said to be *passive* in respect to impressions. Their intellectual faculties do not announce themselves—they are in a state of comparative indifference, and may be modelled by education and surrounding circumstances. It is for such men as these that systems of education should be calculated. It must always be recollected, however, that the minds of men differ as much as their bodies, and that, between the idiot and the sublimest genius, every gradation of the intellectual scale is fully occupied. But each individual has his boundary of elevation, beyond which he cannot rise, however favoured by education or accidental circumstances. If the philosophers of the last century had known these truths, we should have been spared many fine disquisitions on the *equality* of men's intellects, and on the *perfectibility* of human nature.

Our author thinks, and not without reason, that one great defect in education, and which is not a little conducive to the production of insanity, is the greater attention paid to the cultivation of the intellectual powers than to the moral faculties—that is, to arts, sciences, and literature, than to moral and religious feelings. The pride of parents is perpetually urging them to have their children distinguished rather by fine accomplishments than by benevolence, humanity, and all the kindlier affections of the soul.

It is difficult, says our author, sometimes to ascertain whether mental alienation depends on original weakness of the understanding, or a vicious education. M. Pinel relates the case of two orphans, who, being deprived at a very early age, of their parents, were placed under guardians of the most opposite characters. The one was brought up in every kind of effeminacy, indulgence, and idleness—the other was under a very devil, for moroseness, tyranny, and severity. They both became insane before the age of 21, though there was no hereditary disposition to insanity. Thus, extremes meet, and produce similar effects! Many other examples, from undoubted authorities, are brought forward by our author, to illustrate the pernicious effects of indulgence and improper education in early youth, on the mental faculties.

II. *Influence of Political Institutions.* The character, passions, manners, and sentiments of people, are greatly modified by their government, as has been remarked from the time of Hippocrates. To what other cause can we attribute the contrast between the Athenians and Spartans?—the one celebrated for their genius, urbanity, eloquence, and love of the fine arts —the other for their austerity of manners, equity, concord, and disinterestedness. It is to the political institutions of the Romans that we are to attribute those military virtues and superior genius that made them masters of the world. The climates or soils of Greece and Italy have not changed; but the institutions of man have decayed, and with them all those attributes of mind and body by which he was characterized in ancient days.

The power of government on the genius of a people being acknowledged, our author enquires whether particular forms of government may not exert an influence on the production and character of insanity. The most intelligent travellers inform us that under despotic governments there are very few insane people. Our author endeavours to account for the circumstance in this way:—Under despotic governments all the public institutions conspire to prevent the acquisition of knowledge—to stifle the sentiments—and, in short, to completely coerce the passions. The subjects of such governments may almost be said to have no *moral* existence—and consequently the exercise of the intellect, or its organ, the brain, is almost a nullity. The organ, therefore, is not exposed to the causes of derangement. Under republican, limited monarchical, and representative governments, on the other hand, there is every thing favourable for the production of mental maladies. The reasons are obvious. Civilization flourishes under such political institutions. The intellectual powers are developed to their utmost stretch. Emulation, ambition, intrigue—all the passions and propensities are indulged in to excess, and are but feebly restrained by the reasoning faculties. It is under such institutions that the human mind displays all its powers, and pours forth all its treasures. "But, at the same time, the brain, the material organ of these brilliant phenomena, these sublime faculties, is too much exercised, and thus exposed, in common with other organs, to derangements of function or structure, more or less durable, which disturb the reasoning faculties for a time, or annihilate them altogether."

It is thus, by a still more violent, but immediate action on the sensorium, that political commotions and revolutions give rise to mental maladies, and multiply the instances of suicide and crimes.* Men, in such awful circumstances, seem emancipated from the control of reason as well as law, and all the fiercer passions are let loose. Vengeance, cupidity, ambition —nay, even the more generous emotions and passions are carried to such an excess, that the seat of reason is overthrown. But this is not even the principal way in which insanity is produced in revolutionary periods. The vicissitudes of fortune which then obtain in every direction, are too great to be borne. Elevation as well as depression is dangerous to most men. There are few, in fact, who can firmly support either of these changes. The effects of these political commotions, in regard to insanity, were seen and recorded in Peru, after the Spanish conquest—in England, during the civil wars—in America, after the war of independence—and, on a large scale, in the late French revolution. Esquirol has declared that he could trace the ebbings and flowings of this dreadful revolution by the increment and decrement of insanity. The same experienced author has noticed a fact of much interest, as shewing the influence of political institutions in the generation of mental maladies. Formerly the asyla of the insane presented a considerable proportion of their inhabitants tormented with the

* We must take leave to differ, throughout the whole work, from our author, in making insanity, in all its grades and varieties, depend on primary or immediate derangement of the cerebral functions. On the contrary, we know, from long observation and experience, that the causes of insanity, though perhaps primarily acting on the brain, in the form of moral emotions, would but rarely (comparatively speaking) disturb the intellectual powers, were it not for the derangements of function induced in various other organs, as the stomach, liver, &c. which re-act on the sensorium, and greatly exasperate the original morbid impression. But we go even farther, and assert that purely physical causes will disorder certain organs in the body, in consequence of which the brain will be so sympathetically affected as to present all the phenomena of insanity. We are ready to admit, however, that the sensorium is strongly predisposed to the intellectual disorder by the causes enumerated by our author.

fear of demons and beings of a supernatural order; but now this class of insane is replaced by one of quite a different description—men who are haunted with the fear of the *police.* The change is satisfactorily accounted for by Esquirol. Religion and superstition have lost much of their influence over Europeans in general, but the French in particular. The consequence is, that the government must employ other means to keep turbulent spirits in order. What power the clergy may have lost, is now thrown into the hands of the police officers—and hence the change of tenants in the receptacles of the insane.

. . . . . . . .

III. *Influence of Religious Institutions.* But although superstition has lost much of its terrors, and perhaps religion itself much of its benign influence over the minds of men, in proportion as civilization has advanced; yet the sentiments of veneration for our Creator—the hope of rewards, and the fear of punishments in a future state of existence, operate, and will for ever operate powerfully on the minds of men, till their nature becomes completely changed. Religion may be abused, and superstition erected in its stead; but still the worship of a Deity must prevail in some form or other. The devout Gustavus Adolphus, and the sanguinary Suwarrow, equally invoked the Supreme Being to crown their arms with success. Louis the Eleventh and Philip considered themselves as raising the most grateful incense to their God, by the Auto-da-fé, and the tortures of the Inquisition. While, on the other hand, the sublimest philosophers, as Newton, Haller, and a thousand others, have acknowledged their conviction of a Deity, by their admiration of his works. But, in weak minds, superstition, or even religion is sometimes too spiritual to be much discussed or contemplated with safety. Each individual forms his notions of Heaven and Hell, according to the nature of his constitution. The violent, the melancholic, the austere individual forms ideas of the Deity, on the model of his own mind, or rather temperament—clothing the Omnipotent with sentiments and dispositions analogous to his own! It is not strange that the imagination constantly dwelling on such subjects should become deranged—especially when a struggle takes place between the dictates of Nature, and the rigid restrictions of a gloomy superstition. Pinel relates the case of a young woman, who was brought up under very severe religious discipline, and who was afterwards exposed to the temptations of love. The struggle between the affections of the heart and the sense of religious duty deprived her of reason, and in this state she was carried to the Lunatic

Hospital—her incongruous soliloquies betraying the nature of the terrible struggle in her mind. The alarms of conscience respecting supposed crimes and breaches of divine laws are frequently the occasion of mental alienation, and even of suicide! These are some of the evils attendant on religion; but which are not chargeable to it, but to the weakness of our Nature.

"Notwithstanding the privileges accorded to the human race, our nature is so feeble, that those sentiments which are most capable of rendering us dignified and happy, if carried to excess, become the source of our greatest misery."

A missionary, says M. Pinel, by his fulminating declamations, and the frightful images which he drew of future punishments, so worked upon the imagination of a credulous vine-dresser, that the latter conceived himself doomed to eternal fires, and that he could no otherwise prevent the same punishment extending to his family, than by what is called the baptism of blood. He first, therefore, attempted the murder of his wife, and nearly effected his dreadful purpose; but failing in this, he quickly immolated two of his younger children, with the view of procuring them eternal salvation! While confined in prison, before trial, he strangled a fellow prisoner with his own hands—and all under the impression of performing an act of expiation! He was now clearly ascertained to be insane, and was immured in one of the cells of the Bicêtre. Here a long reflection on what had passed, and a profound meditation on the circumstance of his not being executed for so many murders, convinced him that he was the fourth person of the Trinity, and that all the tribunals and potentates on earth were incapable of injuring a hair of his head. In this case, as in almost all others of a melancholy character, the insanity was partial. On every other subject, except that of religion, he was perfectly rational. After ten years of solitary confinement, he became so calm as to be permitted the range of the hospital court—and four more years seemed to confirm the idea of his being now harmless. But a horrible scene was all this time preparing in the monomaniac's mind. Having secreted a formidable weapon, he sallied forth one evening, and first aimed a terrible blow at one of the keepers, which happily did not kill him:—he then cut the throats of two lunatics who came in his way, and was proceeding in his career of blood, when he was mastered and disarmed with great difficulty!

The histories of *suicidal* maniacs, urged to the dreadful deed by religious, or rather fanatical impulses, would fill volumes; and need no farther notice in this place.

We must pass over the chapter on the influence of manners and customs, in the production of insanity. In this, as in all other parts of the work, our author labours to prove that the causes of this terrible affliction act directly on the brain, the immediate organ of the mind. We differ from him on this point.

IV. *Influence of Professions.* Under this head M. Voisin brings forward a curious table from M. Esquirol, shewing the relative proportion of different professions in a mass of one hundred and sixty-four lunatics under the care of the aforesaid Professor. It runs thus: "Merchants 50—military men 33—students, 25—administrateurs et employés, 21—advocates, notaries, and men of business, 10—artists, 8—chemists, 4—medical practitioners, 4—farmers, 4—sailors, 3—engineers, 2. Total, 164." The observations of M. Foderé support the above proportions of insane in the different professions. On examining various asyla, M. Foderé found that merchants and soldiers furnished the largest proportion of insane in all these establishments. In accounting for the fact of merchants being the most numerous of all, our author draws a very unfavourable picture of the mercantile character—which he depicts as "destitute of candour, delicacy, or probity." "Sans bonne foi, sans delicatesse, et sans probité." This demoralizing effect of a mercantile profession he attributes, with M. Foderé, to the love of lucre, which swallows up or annihilates all the more noble and elevated passions and sentiments of man. This avarice, or desire to accumulate riches, becomes so imperious as to justify all the means which can be put in practice to effect the object. "We are arrived," says M. Foderé, "at this point, that we esteem nothing but property, and consequently we do nothing but with the view of making money. All our most cherished affections are submitted to a cold-blooded calculation, in the acquirement of wealth."

Such is the moral picture drawn, which, however, is hardly more applicable to mercantile than to other classes of society. Money is the medium through which all real or imaginary good things in this life are obtained, and merchants are not much more fond of these good things than their neighbours. But let us take a view of the pathological effects of trade, as sketched by our author.

"The chances of speculations, which keep the mind constantly on the stretch, and which, in a moment give or take away a fortune—the life of indolence and voluptuousness in which merchants plunge after a life of tumult, agitation, and activity, explain the frequency of mental maladies among this class of society."

These circumstances undoubtedly lead to hypochondriacism and insanity, in numerous instances, but not, we think, in the way which our author imagines. Inactivity and intemperance, or at least voluptuousness, after an early life of labour, are surely not circumstances likely to act *directly* on the brain. They are far more likely to act on the stomach and other organs of digestion, and thus to affect the brain secondarily. And if M. Voisin had observed as carefully the actual state of things, as he studied in his closet, he would have come to this conclusion, and not taken up the exclusive doctrine which he maintains.

In respect to the military, our author is not more complimentary than to the merchants.

"The wandering life of soldiers—the imperious yoke of discipline under which they are necessarily placed—the pride and vanity which perpetually spring up in their breasts amidst this subjugation—their licentiousness in all other respects except in military duty—the alternations of heroic devotion and idleness—the disasters to which they are exposed by flood and field—their alternate privations and unrestrained indulgences—the ingratitude which they too often experience in return for their services—and finally, the calm and monotonous life of peace after the tumults of war—these are the prominent causes of the multiplicity of suicides and maniacs which we see among the military classes of society."

This we grant; and still we maintain that all these causes, moral as well as physical, are operative on various other organs as well as the brain. And even when they act primarily on the brain (moral causes for example) the disturbance of function produced thereby in the organs of digestion, the heart, &c. re-acts with far more effect on the sensorium than the primary causes. Attentive observation, indeed, would lead us to the conclusion, that moral causes rarely produce insanity, or even hypochondriacism, merely through their agency on the sensorium. It requires, in general, a wider range of sympathetic disorder to take place before the seat of judgment is shaken. This view of the case is strengthened by the treatment. Let us take, for example, a military man who has been exposed to all the causes above-mentioned, and who finds himself passed over, unpromoted, and unemployed at the termination of a war. He becomes deranged. How do we treat him? By secluding him, and keeping him on a rigid system of diet and discipline. Surely this plan is not calculated to prevent reflection, or lessen the chagrin by which he was harrassed. No. But it cures the disordered state of other organs, by the system of abstemiousness and regularity enjoined—and when these disorders are cured, the sensorium, in a great majority of cases, recovers its healthy functions. The fact is, that in most cases of mental chagrin, arising from disappointed ambition, the individuals fly to drink, for the purpose of drowning reflection—and this system soon brings on, or accelerates the accession of insanity. The plan of treatment above-mentioned strikes at one of the great roots of the evil, and is thus generally successful.

Among the professions which predispose to, or occasion insanity, must be reckoned the sedentary. In these the muscular system is left unexercised, and the digestive apparatus becomes deranged. "To live," says Rousseau, "is not merely to *breathe*. It is to *act*—to make use of our organs, our senses, our faculties—in short to exercise every part of the body, and every power of the mind." How few, in civilized life, fulfil this precept! The physiological explanation which our author gives of the effects of sedentary habits is as follows:

"This inaction of the muscular system, and of the agents generally of our external relation with the world around us, lessens the expenditure of nervous influence on those organs and parts, in consequence of which the equilibrium of the sensorial power is disturbed, and a concentration of it is produced in the principal focus of sensibility—the brain."

We would view the matter somewhat differently. Sedentary habits induce general debility, not only of the muscular system, but of the digestive functions; and this debility is invariably accompanied by *irritability*, not only of the brain, but of the whole nervous system. In consequence of this state of things, a morbid susceptibility to every impression is engendered, and the sensorium is continually disturbed. In this way hypochondriacism and insanity are no doubt occasionally produced.

V. *Influence of Age.* This subject is brought forward by M. Voisin, with the sanguine expectation that it will greatly strengthen his favourite doctrine that—"the causes of insanity tend to derange the organization of the brain merely by the exercise of its functions." There can be no doubt, that the periods of life most favourable to the production of insanity, are those when the brain is in most activity. Insanity rarely begins before puberty, when the powers of the mind begin to expand—and it continues to take place, or augments at all subsequent periods, while the mental powers are in full play—and finally diminishes in old age, when the intellectual operations begin also to flag. This statement is correct, but does not in the least militate against the view we have taken of the operation of the moral and physical causes of insanity. The period above-mentioned, for instance, between 20 and 50, is precisely that in which the digestive organs are also most readily deranged from the same causes that act on the sensorium. Hence we have every reason to give them credit for their full proportion of effect in this class of maladies.

VI. *Influence of Sex.* It has been pretty generally observed that a greater proportion of females than males become insane. It is not difficult to account for this, when we consider the greater nervous susceptibility of women—the vice of their education—the crosses and chagrins to which they are exposed—the keenness of their feelings—and the life of celibacy to which they are so often doomed in high states of civilization. An average has been attempted by our author which would make the proportion of insane as ten males to 13 females. There must be great uncertainty, however, in this calculation.

The physical causes of insanity next engage our author's attention; and first, suppression of the menses. On this, as upon all other points, M. Voisin takes but half a view of the subject. That one of the most common causes of suppression of the menses is to be found in violent emotions of the mind, there can be no doubt:—hence, our author concludes that suppression of the catamenia is a consequence and not a cause of insanity. He overlooks the important fact, that the disorder of the uterine system, though caused by the cerebral disturbance in the first instance, becomes itself a cause (on the well ascertained principle, that all sympathies are reciprocal) of disordered function in the sensorium afterwards. Thus we might fairly lay it down as a practical axiom, that whenever a moral cause of disease acts on the brain, it will be followed by a series of sympathetic, or physical causes re-acting on the same organ, and thereby giving double, triple, or quadruple force to the original cause. Did M. Voisin never observe that a moral emotion, of a sombre kind, would disorder the stomach, and that this disorder would produce pain or other affection of the head? If he did not, he is not an observant practitioner—and if he did, he ought not to suppress this fact, because it militates against his own doctrine. The following case, which M. Voisin brings forward, is really more conclusive against him than for him.

"A young woman, 27 years of age, of amiable character, and very sensible, experienced a severe domestic affliction, and a sudden suppression of the menses then flowing. At the two following periods there was a slight accession of delirium; but at the third, her ideas became extremely deranged, and she was under a conviction that she was possessed by a dæmon, who was constantly suggesting some diabolical thoughts in her mind. She went from house to house imploring her neighbours to exorcise the devil. Her face was flushed, her eyes sparkling, and she complained of a strangling sensation in her throat, the workings, she believed, of the evil spirit. She had recourse to amulets, crosses, and images, to protect her from the devil; but all would not do, and in this state she was admitted into the Salpêtriére. The governor spoke to her in a firm tone, and assured her that no dæmon dared to shew his face within that asylum. He took her under his protection, and gave her a dress that was to prove a complete spell against all machinations of the evil one. The change for the better was soon visible, and in three months the patient went out quite ashamed of ever having been afraid of the devil. At each period of menstruation, however, she was troubled with symptoms of a return of her malady, but they generally went off in a few days."

Now granting, for the sake of argument, that the explosion of insanity, and the suppression of the catamenia, were so simultaneous in the above case, that there was no reason to consider the former as an effect of the latter: yet the partial return of the hallucination at each subsequent epoch of menstruation, clearly shews the influence of this process on the cerebral functions. For our own parts, we believe that, had not the moral causes operated so strongly in this young woman as to interrupt the uterine function, no insanity would have taken place. These moral causes would not have been sufficient to disturb the intellect, had they not had the powerful co-operation of the physical cause, in the form of the suppression of the catamenia. This holds good in most other cases. Mental anxiety will go on, without mental alienation, till some important function, as that of the liver, stomach, or uterus, becomes deranged, and then the cerebral functions are unable to withstand the double shock of moral and physical causes; and insanity, hypochondriasis, or some other form of intellectual aberration is the consequence.

### HYSTERIA.

In the investigation of this complaint, our author adheres to the same exclusive doctrine, and maintains, that the primary cause, and immediate seat of hysteria, is an idiopathic affection of the brain. But the cases which he brings forward refute his own doctrine; at least so far as to prove that the uterine system is intimately connected with this strange complaint in many instances; and that without this uterine disorder, or one of some other organ besides the brain, the disease would rarely take place. We shall present one or two cases taken at random.

*Case* 1. " A young lady, 17 years of age, of great susceptibility, became deeply in love with a young gentleman; but concealed her sentiments, at the expense of great violence to her feelings. One day, while in the midst of the menstrual process, she observed her sweetheart paying his addresses to another lady. It was with the utmost difficulty she could suppress the demonstration of her mental agony. The consequence of this internal struggle, however, was a sudden stoppage of the menstrual secretion. She now turned deadly pale—her features became quite altered—she fell into a state of insensibility, and convulsions followed. These subsiding, she alternately cried, laughed, tore her clothes, bit her arms, and complained that a ball was lodged in her throat and strangling her. At the end of the paroxysm, she discharged a large quantity of limpid urine. Several paroxysms of this kind succeeded, and the narrator (M. Louyer Vilermay) was called in. He prescribed antispasmodics, and the usual remedies resorted to in hysteria, but without success. The fits returned, during a period of six weeks, without mitigation. At this time, Vilermay drew from his patient the secret of her mental distress, and endeavoured to console her by arguments; but these also failed. Two months had now elapsed, and no cessation of the hysteria, nor appearance of the menses took place. Vilermay applied a dozen of leeches to the vagina. Almost immediately afterwards the catamenia came on, and the hysterical paroxysms gradually diminished, and finally disappeared."

What does this case prove? To our minds it appears unquestionable, that the moral emotions were the immediate cause of the catamenial suppression, and that this catamenial suppression then acted as the principal cause of the hysterical paroxysms. That the moral cause alone, would not have been sufficient, in this case at least, to induce the hysterical convulsions, we have every reason to infer, because, when the physical cause was removed—when the uterine function was restored, *the moral cause still remaining*, the young lady was relieved from the hysteria. We think it is hardly possible to have a clearer proof of the positions we have laid down, or a more unequivocal catenation of cause and effect, than in the ratio symptomatum of this young lady's disorder.

We shall only cite one more case, taken also from Vilermay's excellent work on Nervous Diseases.

*Case* 2. " A young woman, 21 years of age, of good constitution, and very regular in her menstrual periods, became ardently attached to a young man, and the attachment was reciprocal. But the parents of the young lady resolutely opposed the connexion. *From this time her health became slightly deranged, and menstruation irregular.* ("Dès lors on remarqua un léger dérangement dans sa santé ; et le cours de menstrues fut irregulier.") During the space of six months, the lady experienced several accessions of hysteria, accompanied by convulsive movements, sense of strangulation, globus hystericus, and a feeling of formication in the region of the uterus. ("Fourmillement vers l'uterus.") Some time after this, she accidentally saw a letter of her lover in the hands of her parents, which they refused to shew her. She was now suddenly seized with a paroxysm of hysteria more violent than any that had preceded. Bleedings, blisterings, and various means were used, but it was seven or eight days before she completely recovered from the attack, remembering only vaguely the sufferings which she had experienced. Her parents still continued obstinate in preventing the prosecution of the courtship, she was taken on a tour, and gradually recovered serenity of mind and health of body."

Every one must see that this case merely shews the influence which the mind exerts on the body. The crosses in love deranged the general health, and rendered the function of the uterus irregular. Then hysteria became developed—and a sudden and violent emotion of the mind, in this state, produced a paroxysm to which it is difficult to assign a name, but which was no other than an aggravated form of hysteria. Moral and physical remedies were applied in the amusement and exercise of travelling, and by these the mental and corporeal maladies were cured.

We would be the last to deny the influence of moral causes in the production of disturbed function of the brain and nervous system; but we would look a little farther than our author does, and trace the sympathetic associations by which these original impressions on the sensorium are aggravated and multiplied. We do not deny that a very violent mental emotion may, at once, so impair the functions of the brain as to induce insanity; but we maintain that this is a comparatively rare case; and that, in the great majority of instances, the intellectual disorder is the result of a combination of moral and physical causes, acting and re-acting on various organs of the body.

The subjects of Nymphomania and Satyriasis next occupy our author's investigations. We shall not dwell long on such disgusting and distressing topics. We believe that, throughout the organology of Gall and Spurzheim, that point is the best established by facts, which places the organ of physical love in the cerebellum. No one can contemplate the dreadful effects of nymphomania and satyriasis with attention, and not come to the conclusion that the irresistible propensity depends on organization, though it may be increased by indulgence and by the want of those restraints which moral and religious principles impose. We consider it highly probable, if not quite certain, that this peculiarity of organization leading to the abovementioned diseases, is not confined to the mere organs of generation, but that it has a deeper root, in the nervous system itself—and it would appear, from numerous observations, to be in the cerebellum. At the same time, we believe it to be an established fact that, in such unhappy cases, the organs of generation are more than usually developed, and, consequently, although the physical impulse takes its origin from the brain, the theatre of its action in the genitals becomes a participator in the malady—indeed, a *particeps criminis*.

Our author has brought forward, from various authorities, a number of remarkable and melancholy instances, where nymphomania and satyriasis were developed at a very early period—at three years of age, for example, and where, of course, the genital organs, *in themselves*, could not be supposed to be in activity. The impulse was, in every probability, from organization of the brain.

It is asserted by our author and by Gall and Spurzheim, that, in all the cases of nymphomania and satyriasis, there was observable a remarkable development of the cerebellic region—and that in the busts and pictures of all those personages celebrated in ancient and modern times for salacity, the nucha is remarkably prominent. But, while our author contends that original conformation of the cerebellum gives the impulse to the diseases in question, he equally contends that various moral and physical causes may so develop this organization of the brain, as to produce the disease where it would not have existed, without these incentives. Among the causes of this development, he places novel reading—balls and plays—sedentary habits—erotic conversations—music—the contemplation of statues and prints. The histories of cases placed on record by respectable authors, shew these causes as having been in operation in the majority of cases.

It would have been very desirable that our author should have produced some instances of cure, on the principle which he espouses. He informs us that, in cases of nymphomania and satyriasis, leeching and blistering the nucha, will have more effect than all the other remedies we can administer. We should like to see this practice put to the test.

We shall pass over the long chapter on hereditary disposition to insanity. This is no longer a matter of doubt among practitioners and attentive observers, though it still furnishes themes for theses and essays among the noviciates of the profession. Dr. Voisin considers mania as a disease of structure, or at least of organization, in the brain; and if so, peculiarities of structure favourable to the impression of the moral causes of insanity may be transmitted from parent to progeny, in the same sense that the disposition to gout and phthisis is rendered hereditary.

POST MORTEM RESEARCHES.

The advocates of a particular doctrine, or a favourite hypothesis, are remarkably fortunate in discovering proofs in support of their subject, wherever they turn their eyes. Dr. Voisin fearlessly comes to what might be considered the *experimentum crucis* of the brain being the *primary* seat of insanity. He observes, however, that from attention to what occurred under his own eyes in the Salpêtriére, and from the comparison of published records, it would appear that, in the bodies of the insane, we often find chronic inflammation of the thoracic organs, with the various consequences of this state, adhesions, effusions, hepatization, &c. Indeed, he says, "the lungs are rarely sound." In many females who had died at the Salpêtriére in a state of insanity, but who had scarcely exhibited any symptoms of phthisis while living, there were presented the most unequivocal ravages of the disease on dissection, such as tubercles, excavations, abscesses, indurations, &c.

In the abdomen, the mucous membrane of the intestines was very often found injected, thickened, or ulcerated, in different parts of its surface. The obliquity of the colon, as observed by M. Esquirol, was frequently noted by our author. The liver was very generally altered from its normal state, in various ways. There were seldom any organic lesions of the uterus or the ovaries.

The brain, of course, engages our author's attention, and he gives us the results of M. Georget's researches, as stated in the New Dictionary of Medicine.

Morgagni made but seven or eight dissections of subjects afflicted with insanity; but in these he noticed several organic lesions. In most instances, he found the cerebral substance of the hemispheres sufficiently firm, while that of the cerebellum was soft. He also noticed vascular turgescence, adhesions, infiltrations, &c.

Greding has noticed thickening of the cranium in one hundred and sixty-seven cases out of two hundred and sixteen. He found, in a considerable number of cases, hydatids in the plexus choroides—and, in 51 out of 100, softening of the brain. In many of these, epilepsy was combined with the insanity. The other lesions which he mentions, were such as have been already stated.

Dr. Haslam assures us that insanity, in every instance, is accompanied by organic alterations in the brain, and in these alterations he places the proximate cause of the disease. Dr. Haslam has given 30 dissections, shewing various kinds of structural changes in the encephalon.

In M. Esquirol's examinations, there is scarcely a species of disorganization which he has not found in the brains of the insane. It is needless, therefore, to repeat them. Spurzheim avers that he *always* found organic alterations in the heads of insane people. M. Georget dissected a great number of brains, and his experience is conformable with that of the authors above-mentioned.

M. Voisin asks triumphantly, after this display of evidence, if pathological anatomy can be said to be a sterile ground in respect to insanity? But he does not seem to appreciate the difficulty of determining whether these structural changes be the causes or the effects of insanity. The pathologist has the same difficulty to contend with in insanity that he has in fever. In most cases where death takes place, we find the structure altered in both diseases: but who can say that the commencement of the structural alterations was exactly coeval with the symptoms of insanity in the one case, and fever in the other? If the phenomena of madness display themselves before the structure is changed, we have no right to infer that the change of structure was the cause of the mental derangement. Besides, we every day see all these changes in the brain and its membranes, without any symptoms of insanity. Let us take the stomach, for example, and see how long its *functions* may be deranged by improper food and drink, without any change of its structure. It is, in some respects, the same with the brain. A variety of moral and physical causes may conspire to disturb the intellectual *functions*, and produce mental alienation; and yet the structure of the brain may not be altered. We have a right to infer this last circumstance from the number of recoveries which we every day observe, after the most unequivocal attacks of insanity, and where it would be absurd to suppose that organic disease in the brain or its membranes had taken place. It is, therefore, far from being so clearly proved, as Dr. Haslam and our author suppose, that the organic changes found on dissection are the causes of the mental aberration. We are far more inclined to agree with Pinel and Esquirol, than with Voisin and Haslam, that disordered intellectual function of the brain may go on, for some time at least, the same as disordered function of any other organ, before the structure becomes sensibly changed—and, consequently, we believe the lesions which we find in the brain or its coverings, after death, are effects of long-continued derangement of function. This is what we see in other organs and parts of the body, and we know of no reason why the brain should be exempt from the laws which regulate other portions of our physical frame. The following passage in M. Georget's work on mental derangement, is, we apprehend, much more correct than the doctrines of Voisin, Haslam, and Lawrence.

"In short, the physical disorganizations which are presented in the heads of the insane, are not constant or uniform in the majority of cases, and the very same changes of structure are found in the heads of others who have never been insane; we must, therefore, consider these changes as the *consequences* of the primary lesion of function, (the precise nature of which we are unable to ascertain) and the *causes* of various chronic nervous affections, of a secondary nature, which we find so frequently among the subjects of insanity."

This doctrine will be found more applicable to practical purposes than any other. When a case of insanity presents itself, we should not look exclusively to the brain; for, even if purely moral causes have disordered the functions of the encephalon primarily, there will necessarily be various other functions disordered in consequence, and these disordered functions, say of the liver, stomach, &c. will so re-act on the brain, as to greatly increase the mental malady. It is not, therefore, by bleeding and blistering the head *alone* that we can hope to treat insanity successfully. We must endeavour to withdraw, if we can, the moral causes—and, if we cannot do that, we must strive to obviate their physical effects on the brain and on the other organs.

If we could confine the operation of the original causes of insanity to functional derangement, the disorder would ultimately wear itself out, and the intellect would be restored to a state of integrity; but, unfortunately, changes of structure too often supervene on this *disorder*, as well as upon others, and then the *disease* is confirmed. One of our chief objects, then, in the treatment of insanity, is to prevent change of structure in the head or elsewhere; and, for this purpose, we must have recourse to leeches, blisters, cold to the head, spare diet, and seclusion. When any other organ appears deranged in its function, we must pursue those means which are calculated to restore healthy function, and prevent organic disease. In these few precepts, we believe, the whole art and mystery of treating insanity (as far as medicinal means are concerned) may be concentrated. It is true, the physical treatment of insanity is not much in vogue; the moral means being those principally trusted to—especially *isolation*. We are fully aware of the great importance of the removal of the insane from the sight of all friends and acquaintances, without which, indeed, a cure can hardly be expected; but we are quite confident that much may be done by those remedies, which prevent or correct determinations of blood to the head, and disordered functions in other organs.

The work of M. Voisin is more distinguished for interesting extracts from other writers, than by original materials brought forward by the author. Indeed, four-fifths of the work are composed of excerptæ from the best modern writers on the subject of insanity—and, perhaps, this very circumstance renders it more valuable. Readers, however, must be on their guard against the exclusive doctrine which M. Voisin endeavours to support, and from which all his arguments take a colouring. No doubt, too, the extracts are not quite so impartially taken as might be wished, for the reasons which we have already stated. We cannot help recommending the work to the libraries of those who have the charge of institutions for the insane.

---

It now becomes our duty to give some account of the second work at the head of this article. Dr. Knight informs us that the present publication is "founded on the notes and observations, the result of a personal examination of the symptoms of insanity, in the cases of about 700 lunatics, which examinations were carefully made, and very frequently repeated during the progress of the treatment." It may be also noticed, that Dr. Knight drew up his work in a rough state before he examined the writings of others, in order that his own judgment might not be biassed in the conclusions which he drew. On turning to authors, he was alternately gratified and astonished at the coincidences and disagreements which he there observed. This however, could hardly be expected to be otherwise. One of the greatest novelties in this little work, is a monograph statement of the emotions, sensations, and ideas of a person during the progress of insanity.

"The narrator is a gentleman of considerable talent, and liberal education. During his convalescence, he ransacked my little library, and having soon exhausted all he chose to read, I suggested to him to write an account of his ideas, &c. during his late derangement; having previously remarked that he remembered, with the accuracy of a sane person, most of the occurrences that were at all likely to arrest the attention; he most cheerfully consented." vi.

Of this curious and, we believe, unique document, we shall speak presently. But we must first glance at the prominent features of the work itself.

In respect to the *proximate cause* of insanity, our author is inclined to coincide with Cullen, in viewing it as a peculiar, or rather a disordered state of the nervous system. He has no hesitation in declaring his conviction, that—" in every case of deranged intellect, the disease proceeds immediately from corporeal disorder." But the exact nature of this corporeal lesion our author does not pretend to know. Dissection, he thinks, has thrown no light on this subject. He has examined a great number of the bodies of the insane, and although he has not found any morbid appearances that are not frequently met with in bodies where insanity had never been manifested, " yet he thinks he has observed that there has been greater turgescence of blood-vessels, and more copious effusion of blood —in fact, greater evidence of an excessive vascular action in the brains of insane subjects, than would have remained under the same system of depletory treatment in the same subject, had he been sane, and died sane." This observation may be correct, and it may be practically useful in guiding us as to the treatment; but it gives us little light as to the proximate cause of insanity; for Dr. K. need not be told that turgescence, even to bursting of the vessels, is every day seen, without the slightest symptom of insanity. The peculiar condition of the nervous system—to which we may apply the term *irritation,* for want of a better,—must, we think, precede this vascular turgescence, the same as it does in inflammation; and is, therefore, more entitled to the character of proximate cause than the vascular phenomena.

Dr. K. admits that, whatever this susceptibility to insanity may be, it is often hereditary—" and it is equally true that some families have *visible* peculiarities of structure that descend from generation to generation." When a fortuitous concurrence of circumstances has induced mental derangement in an individual not having this hereditary taint, he has never known an instance of the disease being thus transmitted to the children. But then the question recurs, how does it ever become hereditary? It must surely begin somewhere; and more likely in a case of this kind, than where the parent has never experienced an attack of the disease. From the subject of hereditary disposition, Dr. K. starts off, in a very abrupt manner, to an attempt at explaining " *the cause of sensation produced by the action of reflection.*"

" It has been well observed by an eminent moral philosopher, that when we reflect, or are endeavouring to recollect, we are conscious of some movement—of an act, or series of acts, within the cavity of the cranium, as though hunting about among our thoughts.* And who has not, when tossing on the restless couch, anxiously courting sleep, experienced the tranquillizing sensation of sleep stealing over him, felt his brain gradually relieved from the busy throng that has crowded there, felt as though its energy had relaxed, and the power that had *pressed* it to action had ceased—that is, felt he was tranquilly falling asleep, and then suddenly been roused by all the sensations of thought renewed, in fact, by a return of the *pressure?*" 6.

From these considerations, Dr. Knight is led to the conclusion, "that the intellect is *developed by pressure*, which, in the sane, can, for the most part, be exerted at will; but, in the insane, acts independently of any voluntary movement." We are sorry Dr. K. should have put on record such an awkward attempt at solving a problem, that no human power has solved, or can ever solve.

Insanity is thus defined by our author:—"*A confusion in the intellect, with some degree of correctness of perception and consciousness—the confusion being frequently, in the early stage of the disorder, manifested more by actions than words.*" We never attached much importance to definitions of diseases, since they are all imperfect. They are attempts to squeeze into a line or two a group of characteristic features of a complaint requiring as many pages for their description. It is evident that all the foregoing symptoms may be produced by a blow on the head—nay, by a disorder of the stomach, and where there is not an atom of insanity in the case. The definition, therefore, is of no use whatever. Dr. Knight has sketched a few examples from his note-book, illustrating the errors of perception, conception, memory, &c. some of which are rather curious. Thus, a man, Henry Williams, conceived himself to be a fat sheep, and in every rosy-faced man who visited the asylum, he perceived a butcher, who was about to lead him to the slaughter-house. The consequences of such an hallucination may be readily imagined, and were sometimes even ludicrous. A jolly justice of the peace frightened this poor lunatic more than any other personage who visited the asylum.

Another man was thoroughly convinced that he had a mouse in his throat, and was always soliciting the bye-standers to pull it out by the tail, which he said was at the root of his tongue.

Our author criticises a passage which we quoted in this Jour-

* "See Aristotle de Memor. et Reminiscent. c. i. p. 680. Dr. Gillie's New Analysis of Aristotle's Works."

nal from Esquirol on Insanity. That experienced physician mentions, as one of the peculiarities of insanity, the loss of that delicacy which characterizes the words and actions of the female sex. It was never meant to apply, even generally, to the insane, but as shewing how far the natural sentiments of an individual may be sometimes perverted by the malady. Whoever has seen much of insanity, will justify the observation of M. Esquirol. The remark, however, does not apply to monomania, where there is an hallucination on only a single subject. It applies more particularly to general acute mania, of temporary duration. In such instances, we have heard *ladies* make use of language, that really surprized us as to the where and when they could have learnt it!

The pusillanimity of the insane has been noticed by all writers. The following observation of Dr. Knight's is judicious.

"The inevitable result of insanity is infirmity of purpose, and the equally inevitable result of infirmity of purpose is its near relative, pusillanimity; to which I may add, discontent—the besetting misery of the insane: and the immense vituperative mass that has been said and written, of the cowardice, ingratitude, dissimulation, and especially discontent of the insane, may, I humbly conceive, be much better, because more fairly, more clearly, and briefly, comprised in this sentence: Insanity begets infirmity of purpose; infirmity of purpose begets pusillanimity and discontent. I have frequently noticed, that the first symptom of returning sanity, has been a diminution of discontent." 14.

M. Esquirol has observed, that the passions (by which he generally means moral causes and their effects, as grief, love, fear, jealousy, &c.) have a vast influence in the production of insanity. Dr. K. we imagine, does not clearly comprehend the meaning of M. Esquirol. He contends that the passions are manifested more as the effects than the causes of insanity. This is doubtless the case; but still, as long as moral emotions are acknowledged to be the frequent causes of insanity, the observation of M. Esquirol will be considered as based in practical observation. That Dr. K. misunderstands Esquirol, and confounds the play of the passions with violence and irritability of temper, will be rendered probable by the following passage.

"As confirmatory of this fact, I may, perhaps, with propriety, here state, that of about 120 insane women, who were at the same time under my care, on an average, *not more* than *three or four* were confined, or coerced, *on account of being violent*, and not more than ten or twelve, on any account whatever; and of about 130 men, who were in the same establishment, on an average, not more than *two or three* were confined or coerced, *on account of being violent,* and not more than ten or twelve on any account whatever." 26.

On the influence of moral causes in the production of insanity, our author makes many judicious observations. We agree with him, that *moral causes alone* are seldom sufficient to produce insanity; but these moral causes having once induced disorder of function in the body, become powerfully assisted by their own physical effects, in disturbing the seat of reason. The following passage is worthy of notice.

"Insanity, like some other diseases, may have for its origin a moral, or a physical cause; either an affection of the mind, or a disorder of the body. The moral impulses, however, very rarely produce insanity, and this is also the case with regard to religious feelings. I come to this conclusion, because of nearly seven hundred cases of insanity that I have sedulously treated, I have only *once* ascertained, with that clearness, which I think the importance of the subject required, namely, unquestionable proof, that either a religious or a moral cause produced the disorder. 'Tis true, I have frequently been informed, that this and the other person became religiously insane, through following some sect not connected with the narrator's persuasion; but when it has been possible to get an intelligent history of the person, I have uniformly found, that the individual had betrayed at least equivocal symptoms of insanity, and that derangement of the mind, though not palpable, had obviously existed, before he became a raving devotee; and doubtless from this state of mind, has arisen that proneness to change his mode of worship, so frequently noticed *in him*, who is what is termed religiously insane—not that the change in his mode of worship has caused the insanity, as has been, I think, erroneously stated; but that the incipient insanity has caused the fickleness in devotion, together, probably, with an unusual fervour, which, if urged on by an erroneous zeal, as it too often is in these cases, may, and doubtless very frequently does, assume the impression given to it—whether that be of the usual gloomy cast, or whether it be of a lively and amorous character, as I have sometimes seen it. As this latter is rather a rare species, of what is termed religious insanity, I shall make a few extracts in illustration of it, from one of my journals, which I fortunately possess.—October 11th, 1823, Elizabeth A. a lusty young woman, admitted this day.—Oct. 17th, she pointed out to me the 5th chapter of Ephesians, and said she was reading an exhortation to love, her manner and expression of countenance corresponding to the sensual import of the passion. Another time, she pointed out the 2nd chapter, 5th verse, of Solomon, and with voluptuous expression quoted these words:—'He took me to the banqueting house, and his banner over me was love.' She was always talking on religious subjects; and she had hymn-books, wherein the passion of love was too warmly pourtrayed, as I conceive, to be suited to its professed object: many of these she had by heart, and would sing to the tune of profane airs, with all the expressions of an amorous passion.

"Similar observations may be made respecting the other passions, owing their origin to corporeal disorder. Terror, however, claims particular notice, as it has, doubtless, by its violent and sudden action, frequently produced instantaneous insanity; and perhaps more frequently than all the other passions combined; and insanity from this cause is the only one resulting from the passions which I have satisfactorily ascertained. It was shortly this.—Some household concerns induced a mother to leave her infant without any competent person to attend to it. On her return, the shrieks of the infant made her rush to its protection; it was writhing on the floor dreadfully burnt: she sprang forward, and with her hands crushed the flames. Here was a horrid spectacle!—the infant was dead,—the mother deranged." 35.

We think the case of the erotic, Elizabeth A. bears us out in our observations on the temporary loss of delicacy which the insane female occasionally experiences. In our author's anxiety to prove that religious feeling, and other moral emotions, are rarely the cause of insanity, he seems to overlook what we have so often stated, that these moral causes produce the physical lesion, and the physical lesion the disturbance of intellect. In this sense, although moral emotions and the passions are not the *immediate* causes of mental derangement, they are still entitled to the denomination of *primary* causes.

*Physical Causes.* Our author has scarcely ever found insanity "unaccompanied by one or more corporeal diseases." We scarcely know what to say to the following observations.

"The skilful medical practitioner will generally be able, after one or two careful examinations, to detect the seat of considerable bodily disorder, which most probably has given origin to the derangement of the mind; and for this purpose he should avail himself of every means in his power, not relying on the statements, either of the patient or his friends, but by manual examination he should ascertain, if there is any local cause of pain, or constitutional irritation, as congestion in the liver, or partial obstructions in the bowels, or disorders of the heart, and of the lungs especially. *No symptom should escape the severest scrutiny; and by a cautious induction, it is probable, that the true cause of all this frightful disturbance may be ascertained.* But, in his examination, the physician must constantly keep in mind these anomalies. Though labouring under an attack of pneumonia, the lunatic will make a full inspiration, and tell him he feels no pain. With every evidence of determination of blood to the head, and intense head ache, he will tell him he was never better in his life, &c. Whatever this disturbance may be, the general principles of medical practice are applicable, with one caution respecting blood-letting, before alluded to, and subsequently to be explained. What I have said refers to the remote cause, and its effects only: as to the proximate, or immediate cause, I cannot venture to speak with that precision the importance of the subject requires; but I am persuaded, that, as there are many remote causes, so there is more than one proximate cause; and, as I have already stated, that I believe the nerves to be the chief immediate cause, by their diseased action, so I also believe, that constitutional defects, producing organic lesion of the brain itself, or its membranes, are also proximate causes; and I doubt not that the diseased action of the nerves, particularly those distributed to the carotid arteries, and parts more immediately connected with the brain, create an increased action of those vessels, and thus produce a determination of blood to the brain, which, by Drs. Arnold, Cox, and Mayo, has been itself considered as the proximate cause of insanity. *Practice* does not give sufficient support to this opinion, and I consider this determination of blood to the head, as the effect only of the *local* nervous irritability: I shall by and bye bring forward some facts to substantiate my opinion." 41.

Dr. K. then cites various authorities unfavourable to blood-letting in insanity, notwithstanding the apparent determination of blood to the head—and this, we think, is a pretty strong proof that the nature of this proximate cause is not well understood—and, at all events, that it is not common inflammation. Practitioners should bear in mind, that a state of irritation in the nerves of a part will cause an afflux of blood to that part, but that this afflux, in itself, is not inflammation, though it may lead to that disease. This is the case in insanity. There is irritation of the brain, whether idiopathically or symptomatically produced—and a long continuance of this irritation may lead to inflammation and its various consequences. It is in this way that organic changes in the encephalon or its membranes are effects and not causes of the mental derangement. When inflammation or change of structure, however, is added to the original irritation, it is not difficult to imagine that the malady may be increased, and even rendered dangerous as regards life. It is, therefore, with the view of preventing these structural changes, rather than with the hope of thereby curing insanity, that blood-letting should be employed. The moral and medicinal means of soothing *irritation* in the brain and nervous system, are the main points of treatment, as respects the cure of the mental aberration. Much mischief is every day done by indiscriminate bleeding in the high states of insanity, and but little benefit is ever derived in the reduction of furious delirium by the lancet. We fear the following observations are but too true.

"Insanity is very rarely indeed idiopathic, and when it is, my experience leads me to conclude, that it is manifested in very early life, and that it is generally, if not always, incurable. The patients of this class, who have been placed under my care, have evinced an untoward disposition from early infancy, much shrewdness and cunning in their proceedings, with equal hardihood or sullenness, according as they have been treated. The cause remains unknown to me: it may be, and doubtless often is, an organic lesion of the brain or its membranes, which is frequently succeeded by epilepsy—a case perfectly hopeless, and which may continue for years. Medicine has appeared to be of very equivocal use in idiopathic insanity, especially if conjoined with epilepsy; but I have found small quantities of blood, taken either by leeching or by cupping, or by the lancet from the jugular vein or the arm, to have a constant good effect in mitigating the severity, and shortening the duration of the epileptic fits." 46.

But, even in idiopathic insanity, moral treatment, especially under steady discipline by an authorised stranger, may do much towards mitigating, if not curing the malady. Our author illustrates this position by the case of a young gentleman idiopathically insane, and who, when at home, was most violent and irascible, endangering the lives of his nearest relations.

"By a treatment uniformly mild, and uniformly firm, to enforce the rules laid down for his conduct, I succeeded, in a few weeks after his first admission, to make him an obedient and well-behaved youth, even when it was evident that his intellect was considerably affected. The plan adopted was this. The periods of rest, of meals, of exercise, and lastly of study, were fixed and immutable:—when he became refractory, he was admonished; if he persisted, he was instantly compelled to obey,* except as to study, which was voluntary, or only enforced by example and persuasion. After I had once established this system, we were the best friends imaginable; and at the subsequent times of his being placed under my care, he immediately conformed, so that it was extremely difficult to detect any insanity in him. Lunatics of this class are generally healthy." 48.

Dr. Knight next makes many useful remarks on medicinal treatment, under the heads of *Sedatives*, including digitalis,

* "This presumes, of course, that the act required, admitted of compulsion: As, for instance, if he were required to take exercise in the grounds, and he refused, through mere captiousness, he was conveyed to the grounds by persons sufficiently powerful to make all resistance on his part trivial: if, when there, he would not stand, (which is not a very unusual occurrence) though persuaded and gently coerced, he was speedily removed to a solitary and dark room: if he persisted to kick the door, or was guilty of any extraordinary violence, he was secured by efficient means to a proper place already prepared in this dark room: and if, having no other means of annoyance left, he then persisted to scream and halloo, he was either permitted to amuse himself till he was weary, or another *habitually* noisy, though harmless lunatic, was put in the same room with him; this has silenced him in a very short time, and in less than an hour he has come from coercion a tranquil and obliging person. This is the utmost coercion or punishment I ever inflicted on any lunatic patient." 48.

opium, henbane, &c.—*Cathartics*—*Emetics*—*Alteratives*—*Baths*—*Circular Swing, &c.*

In respect to digitalis, Dr. K. confidently asserts, that this medicine, on its *first administration*, is as decidedly a stimulus as brandy or Geneva. Our own experience does not coincide with that of Dr. K. on this point. He acknowledges, however, that after a few days, digitalis never fails to reduce the pulse either in force or number. Is this the case with brandy or Geneva? When the pulse gains in velocity and loses in power, under the influence of digitalis, it is a sign of its deleterious operation, and it should be discontinued. By due attention to the action of this powerful drug, Dr. K. avers, that he has uniformly found it to exert a beneficial effect in allaying the maniacal paroxysms, and reducing irritability, "exactly in proportion as it reduced the pulse, whatever might be the mental action, whether gay or melancholy." Our author has been obliged, in many cases, to give the digitalis for two or three months in succession, in small doses, as from five to eight minims thrice a day, which has kept the pulse steady, the patient being able to enjoy amusements, exercise, or labour. By omitting the medicine, he invariably found the insubordinate disposition return, with increase in the quickness of the pulse.

Esquirol disapproves of opium and other sedatives in insanity, recommending exercise and regimen as the best somniferents. Dr. K. cites Halloran and others in favour even of opium, when judiciously employed. But hyosciamus is preferred, and properly so, by Dr. Knight, and especially when combined with camphor. In admitting the utility of this last combination, we are still disposed to agree with Esquirol that, as a general rule, exercise and discipline are the best promoters of sleep in the insane as well as the sane.

*Purgatives.* Dr. K. has not found that lunatics require more powerful purgatives than other people. Occasionally, however, they labour under obstinate constipation, and that from hardened fæces impacted in the rectum and lower portion of the colon. Here drastic purgatives are dangerous. Our author has found a suppository, composed of one or two grains of elaterium, five of calomel, and five of gamboge, made up with a sufficient quantity of soap, answer the purpose.

*Emetics* have been occasionally administered by Dr. K. but not stronger than a scruple of ipecacuan, with a grain of emetic tartar.

*Alteratives.* In all cases of long standing, Dr. K. has found the blue-pill a valuable medicine. In many cases he attributes the recovery entirely to it—and he never found it productive of any bad effects. Dr. K. apprehends that mercury, in any form, must be injurious in recent cases of insanity, and where there is much excitement. It may be so, if carried so far as to produce a constitutional effect; but, as a component of aperient medicine, it is quite superstitious to dread the influence of this medicine, whose physiological action seems really not to be understood by one in five of practitioners at the present day. Dr. K. however, is not included in this class. He considers the medicine as safe, when employed as an aperient, in cases of insanity.

*Baths.* The shower-bath frequently relieves the head-ache and irritability, in old cases, when the skin is hot and dry. The tepid bath is very grateful to almost all lunatics, and there are few cases where it may not be usefully employed.

"*Circular Swing.* Dr. K. considers this physical agent as possessing immense power."

"A patient, subjected to its action, is speedily affected with giddiness and sickness, and the peristaltic motion of the whole alimentary canal seems to be excited, and in some instances to such a degree, that the patient vomits, and passes fæces in rapid succession and great abundance, along with his urine. I have found the circular swing extremely beneficial in obstinate constipation, and in dyspeptic complaints accompanied with much acid. Mary Sandiford, a very fine young woman, said to me on the 20th September, 1823,—'Putting me in the circular swing did me more good than any thing else: it threw all the sour stuff off my stomach.' Shortly after this, she recovered, and was discharged well. When patients are very unruly, and at the same time afflicted with either of these ailments, it never fails to be physically and morally beneficial. Apprehensions have been expressed least the use of the circular swing should induce apoplexy: having attentively examined the sources of these fears, I conclude them to be perfectly groundless; nor have I ever seen the slightest reason to apprehend such result, nor do I believe it ever can occur, *if the patient be not in a furious state when put into the swing;* but if he be, the excitement of the mind will counteract the effect of the swing, wonderfully powerful as it is. The act of turning him will exasperate him still more if possible; he will struggle violently, and neither stomach nor bowels be affected, at least for several minutes, if at all; and the visible blood-vessels of the face and neck have become exceedingly turgid by the paroxysm of fury and exertion: but, even in this case, it is more than probable that arterial action is checked. When giddiness is felt, the stomach is speedily affected, and the pulse is lowered both in frequency and strength—a process not likely to terminate in sanguineous apoplexy, the only species to be apprehended. With this single exception, or precaution, I consider the circular swing perfectly safe. It is a machine that should be easily accessible in every asylum for lunatics, *but never used* except under the direction of an experienced physician. I consider the best time for its use is a little before retiring to rest for the night, as the unloading of the alimentary canal, the lowering of the pulse, and the relaxation of the skin, very generally predispose to sound and refreshing sleep. In concluding my report relative to this machine, I shall take the opportunity to declare my conviction, that it might be made extremely useful in general practice, especially in some inflammatory affections of the viscera of the abdomen, and probably in the commencement of some fevers. I refrain from troubling my medical reader with my reasons for this conclusion, as I apprehend he will readily recognise them in the facts and opinions recorded in the preceding pages." 63.

*Epileptic Lunatics.* Lunacy and epilepsy is a formidable combination, generally considered incurable. By the tables which Dr. K. has appended to his work, it appears that one in seven of epileptic lunatics have recovered—that is, eight out of 58. The reader will, of course, be anxious to know the treatment by which even this proportion recovered. The details are registered in the books of the Lunatic Asylum for the county of Lancaster, and may there be seen, but the following is an outline of the methodus medendi pursued.

" I have freely used the Spirit. terebinth. rect. as recommended in the Edinburgh Medical and Surgical Journal, by Dr. Edward Percival, frequently with much benefit, the fits being often suspended from their usual accession, and when returning being less violent. Added to this, I have checked the circulation of the blood, when necessary, with the fox-glove, and aided the stomach and liver with Carbo. sodæ pil. hydr. and columbo, according as the use of these medicines would be indicated in ordinary practice. Nor have I hesitated to give all these in conjunction, or variously combined; for I am long since quite satisfied, that much more can be effected by a skilful combination of various remedial means, than by the most judicious exhibition of an isolated remedy. Simplicity in prescription is a good way to *learn* the practice of physic, but it does not appear to me always the most certain method to attain our object. Various other means advocated as remedies in epilepsy have been used, as the Cuprum ammoniat. argent. nitrat. valerian, &c. but I have seen no benefit result from their use, in which I have been much disappointed, since so many distinguished physicians speak highly of these remedies in the cure of epilepsy. When the general health and appearance of the patient did not forbid, I have taken blood in small quantities, from four to six ounces, from the arm, or, which is better, from the jugular vein, with uniformly good effect in shortening the duration of the fit, and in rendering it much less violent; but this bleeding should not be after the fit, nor during it, but immediately preceding it. In these, as in all other cases attended by derangement of the mind, the bowels should be always kept in an active state, but not purged. Straightening the hands and limbs has very frequently appeared to put a stop to the progress of the fit; and where it can be effected without such violence as to hurt, I have always permitted it, and sometimes advised it. This is a vulgar practice, and, like most other vulgar practices, has, I believe, some truth for its foundation." 67.

### MORAL TREATMENT.

This has been greatly improved in modern times—nay, even within the present century. The following passage is well conceived, well written, and pregnant with good sense.

" It is the daily duty of the superintendent of a great number of lunatics, to sooth the irritable, repress the insolent, cheer the desponding, calm the excited, check the forward, encourage the timid, resist the importunate and petulant, but *carefully* to attend to reasonable requests; for he has daily causes to try; and he must, at one and the same time, be counsel, judge, and jury: and, as lunatic litigants frequently possess great acuteness, and always much irascibility, it becomes no trifling task to reconcile conflicting pretensions. He is, however, importunately called upon to decide, and his judgment must be supported by fair and conclusive, or at least plausible reasoning: or discord, discontent, and suspicion, will speedily supersede confidence and an affectionate respect; for when the matter does not touch upon the peculiar hallucination of the lunatic, he generally pays much attention to, and acknowledges the force of reason. Frequently the quarrels of lunatics do not arise altogether from deranged notions of right, but from the same malevolent passions that beget contentions amongst the more sane part of mankind. Lunatics very generally regard with derision or compassion the hallucinations of their fellows, and permit them to indulge in their eccentricities with the forbearance of the sane. It is a curious and interesting spectacle to see them thus acting towards one another. The proud, however, form a general exception to this rule; rival monarchs rarely agree: the only exception that occurs to my recollection is, where one mighty potentate is always exceedingly amused at the absurdity of any one pretending to regal power without his sanction and authority. The anger which he evinces is manifested in epithets of his sovereign contempt, accompanied with bursts of deriding laughter. Such conduct as this requires no interference, much less expostulation or reasoning; either the one or the other would be only parallel to the administering stimulating drugs to those already in a state of excitation; maniacal fury would probably be the result of both practices. On the other hand, it is a great error to pretend to coincide in opinion with the lunatic, acknowledging his pretensions, confirming his opinions, and saying every thing that may be supposed to be pleasant and soothing: fortunate indeed will be the result if the effect is not absolutely the reverse. The lunatic, for instance, who has thus been confirmed in his belief of his own sanity, at once becomes restless, irritable, and importunate, although he was previously tranquil and contented. I have known this apparently trivial error in moral management produce raging and ungovernable madness." 71.

It has appeared absurd to our author to argue with the lunatic, with the view of convincing him of his hallucination. The peaceable lunatic is at first a tranquil auditor, till, finding *his* understanding insulted by the evidence of *his* senses, he straight becomes indignant at the barefaced assurance of his orator. It is best to let the hallucination go unquestioned and unheeded, and to draw the lunatic's attention to some very different subject, and fix it there, if possible. For various excellent general observations on the moral treatment of the insane, we must refer to the work itself. Under the heads of amusements—labour—religion—music—classification of lunatics—Dr. K. offers many practical observations, which are well worthy of attention.

Dr. K. relates some curious instances of obstinate disinclination to food among the insane. One man, John Booth, aged about 35, fasted fourteen days. "He certainly took no food during this period, and though he had access to water, I believe he never drank any."

"He amused himself by walking in the galleries of the asylum, and very seldom sat, or rested; yet he appeared as equal to exercise at the end of the fortnight as at the commencement. His pulse continued good to the last; his tongue, which was furred and brown at the beginning, had become clean; and his breath, which was very offensive, as the breath of the lunatic usually is, had become as sweet as an infant's. He was generally very haughty and taciturn; but had now become more tractable, and I at last succeeded in drawing him into a conversation. He told me he had not experienced any benefit from eating; that it had frequently made him ill; and that he had therefore resolved to refrain from it altogether. I asked him if his objection extended to medicine also: to which he replied, he would take any medicine I thought fit to prescribe. I told him it would be necessary to drink it in beef-tea, to which he consented. A pint of good beef tea was accordingly sent to him, and he readily took it; and in a convenient time the dose was repeated, and so he was humoured, till his appetite returned, when he again took his food as usual: and finally he was discharged well." 108.

Our author cannot call to mind more than one instance where he was obliged to force the lunatic to take food. The plan adopted was, to shut up the lunatics in their rooms, and leave some food with them. By this plan they took food when it suited their inclinations, and no bad consequences resulted. He believes that force is never necessary or justifiable.

Under the head of "Miscellaneous Remarks," we have some curious information conveyed in a desultory manner. We shall notice only a few of these. Lunatics frequently complain of strange sensations in the skin. Thus, one patient asserted there were slow-worms or threads dragging through his skin—another, that he was afflicted by little gnomes, called crickets, that had very sharp beaks, which they were constantly thrusting into his flesh, tormenting him by day and by night—a third, that one part of his skin burned like fire, whilst another part was chilled, and so on. These phenomena only shew how much the nerves of sense, and, in fact, the sensorium commune, are deranged.

Dr. K. thinks that the wounds of insane people heal more quickly than those of others. It has often been remarked that, in lunatics, serious diseases, as phthisis, for example, are masked by the mental aberration, even till a late period. He has seen instances of hydrothorax, where no dyspnœa or inability to lie down was complained of till the last. "This disease frequently exists among these people, and is frequently overlooked." He has also found bowel-complaints, ranging from simple diarrhœa to the severest dysentery, so very frequent and fatal among lunatics, that, in the year ending June, 1824, no less than 71 of 250 patients were attacked with these complaints. This was probably owing to local causes: but Haslam and others have noticed the same thing.

The last days of the lunatic are often closed with atrophy, unattended by any appreciable local disease. Yet the victim has an extraordinary power of disguising or disregarding his sensations. In respect to appetite, the insane, on an average, would eat twice as much as sane persons, and they absolutely require more food than people in sound mind and body.

Lunatics frequently assert that they see strange sights—not seldom his Satanic majesty, who is uniformly represented by them as of some particular colour, as yellow, blue, green, speckled—but not always black, as one would expect from association of ideas in health.

The insane are so remarkable for emitting a peculiar odour, especially when the paroxysm is on, or coming on, that men accustomed to lunatics immediately recognize it.

"John F——, though a remarkably clean man, was so offensive to the nose, that the servants on opening his bed-room of a morning could with difficulty go in to open his window, the stench was so intolerable; and all his fellow patients complained of him. The smell of lunatics, however, has been for many centuries familiar to those who have paid attention to their treatment, and J. Van. Swieten and Herm. Boerhaave have both noticed a most insupportable smell in opening the heads of lunatics; I have also remarked the same kind of smell, but not by any means insupportable. The smell I have perceived bears a striking similitude to the urine of cats, and not unlike that which I have experienced in the water of patients under treatment with the Digitalis and Pil. Hydrarg. for the cure of Hydrocephalus when those medicines were acting favourably. The smell of the *person*, however, is different: I at this present time have a lady under my care, the smell of whose skin is very offensive, nauseous, and brassy. I am well acquainted with a gentleman, the smell of whose skin sometimes resembles the smell left on the hands after handling new and unvarnished brass, being barely recognisable. All these persons have suffered severe afflictions, and I am therefore disposed to regard these smells as symptomatic of mental disturbance. Since writing the above, I have learnt that the lady was for three weeks, by her own account, deranged in mind." 122.

The insane, especially those who are epileptic, are subject to great restlessness and uproar in winter, and in foggy, close weather. In summer, very fine weather, especially if the wind be high and in an easterly direction, produces the very same effect. Fine fresh weather in winter, and cold wet weather about midsummer, are productive of unusual tranquillity and decrease of paroxysms among the insane and epileptic.

The mistaken notion that lunatics bear cold better than other people has long been obliterated. They cannot bear cold so well as people in health. It is true that the maniac, who imagines that he has urgent affairs of the greatest importance on hand, disregards cold, and being almost always in action on such occasions, he does not suffer from the impression of cold. The same immunity, however, would be experienced by the sane, under similar circumstances. Lunatics are certainly more noisy on a bright moonlight night than on a dark night; "but this is wholly caused by the light." The same has been observed by other writers.

We have been thus led insensibly to such an extension of this article, that our limits will not permit us to give an account, as we promised, of the maniac's autograph, at the end of Dr. Knight's work. The document, however, on re-perusal, appears more curious than interesting or useful. The patient appears to have been a young Irish student in this country, and, sane or insane, his ideas partake of all the vivacity, and even extravagance, of a wild Tipperary youth, inflamed with classical, political, and religious enthusiasm. There are, nevertheless, some good touches in this curious morçeau, and some attempt at unravelling the mysterious state of insanity, which would not disgrace the medical philosopher. But we must stop short—returning our thanks to Dr. Knight, for the pleasure and instruction his work has afforded us.

